# A hemodialysis patient with bone disease after pregnancy: a case report

**DOI:** 10.1186/s12882-019-1603-8

**Published:** 2019-11-21

**Authors:** Hannelore Sprenger-Mähr, Emanuel Zitt, Andreas Kronbichler, Manfred Cejna, Karl Lhotta

**Affiliations:** 1 Department of Internal Medicine III, Academic Teaching Hospital Feldkirch, Carinagasse 47, Feldkirch, Austria; 2 Department of Internal Medicine IV, Medical University of Innsbruck, Innsbruck, Austria; 3 Institute for Diagnostic and Interventional Radiology, Academic Teaching Hospital Feldkirch, Feldkirch, Austria

**Keywords:** Hemodialysis, Secondary hyperparathyroidism, Osteitis fibrosa cystica, Pregnancy, Etelcalcetide

## Abstract

**Background:**

Pregnancy is rare in women on hemodialysis. Recommendations for the treatment of secondary hyperparathyroidism (sHPT) and preservation of bone health in pregnant dialysis patients are lacking.

**Case presentation:**

We present the case of a young woman with end-stage kidney disease (ESKD) due to lupus nephritis, who developed multiple brown tumors while on hemodialysis during her second pregnancy. During her first pregnancy sHPT was well controlled and no skeletal complications occurred. Before the second pregnancy she developed severe sHPT. During pregnancy, dialysis time was increased to 24 h per week, the patient was given oral calcitriol, and the dialysate calcium concentration was set at 1.5 mmol/l. In week 20 the patient complained about bone pain in her left hip. Magnetic resonance imaging revealed a cystic lesion compatible with a brown tumor. The baby was delivered in the 36th week by cesarean section. Further assessment identified multiple brown tumors of her skeleton, including the acetabulum, tibia, ribs, skull, thoracic spine and thumb. She required multiple orthopedic surgeries. Three months after pregnancy, etelcalcetide was started, which brought about a gradual improvement in her sHPT.

**Conclusions:**

This case demonstrates that the combination of pregnancy and severe sHPT in dialysis patients can have deleterious consequences for bone health.

## Background

Practically all dialysis patients have chronic kidney disease-associated mineral and bone disorder (CKD-MBD), with secondary hyperparathyroidism (sHPT) being most frequent. The most severe form of hyperparathyroid bone disease is osteitis fibrosa cystica (OFC) caused by massive bone resorption mediated by parathyroid hormone (PTH). The clinical presentation is characterized by bone pain and swelling, skeletal deformities and fractures. Imaging studies show osteolytic bone lesions [1]. Histologically, multinucleated osteoclasts (giant cells) with tunneling bone resorption, peritrabecular fibrosis and woven bone are pathognomonic findings. Hemosiderin deposition causes the macroscopic appearance of a brown tumor (BT). Current treatment options for sHPT such as phosphate binders, calcitriol and its analogs, calcimimetics, and finally parathyroidectomy, have made OFC a rare finding in dialysis patients [2, 3]. OFC occasionally develops in patients with primary hyperparathyroidism (pHPT). Pregnant women with pHPT seem to be predisposed to OFC, as illustrated by numerous case reports, suggesting that in pregnancy bone is particularly vulnerable to the effect of PTH [4–7]. The combination of severe sHPT in a dialysis patient and pregnancy may therefore be the worst-case scenario for bone health. As pregnancy is still very rare in women of childbearing age on dialysis, data on bone disease in that clinical situation are practically absent and guidance for sHPT treatment and the preservation of bone health in pregnant dialysis patients is lacking. We here report the case of a young woman on hemodialysis, who had two successful pregnancies. After the second one, which she entered with severe sHPT, she developed devastating and refractory OFC.

## Case report

The 26-year-old Caucasian woman had developed end-stage kidney disease (ESKD) at the age of 21 due to lupus nephritis. She was treated with conventional maintenance hemodialysis therapy three times a week. At age 22 she became pregnant for the first time. Pregnancy was first recognized at 23 weeks of gestation. The dialysis schedule was intensified to 24 h per week. The patient was treated with sevelamer. She received no vitamin D or calcium and the dialysis bath calcium concentration was kept at 1.25 mmol/l. During pregnancy CKD-MBD was well controlled with calcium and phosphate levels in the normal range and PTH levels around 150 pg/ml. The baby was delivered in week 32 by cesarean section. The premature child was small for gestational age with a birth weight of 1735 g, and a length of 43 cm. Apgar score was 7/8/9. The mother breastfed the newborn for only a few weeks.

During the following three years the patient developed severe sHPT with gradually increasing PTH levels to around 1500 pg/ml, primarily because she refused to take any oral medication such as phosphate binders, calcitriol or cinacalcet.

**Fig. 1:**
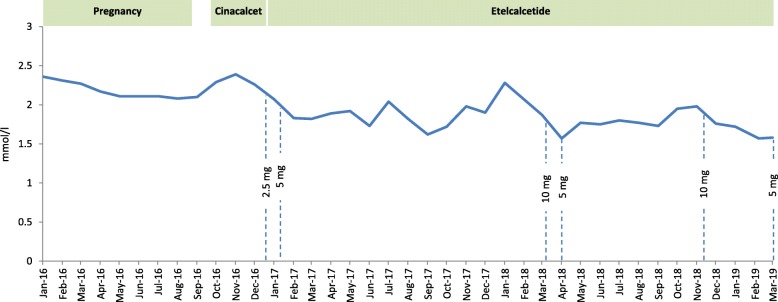
Monthly serum calcium levels starting with the seond pregnancy pregnancy. Calcium was normal during pregnancy. Under treatment with etelcalcetide mild hypocalcemia was present

**Fig. 2:**
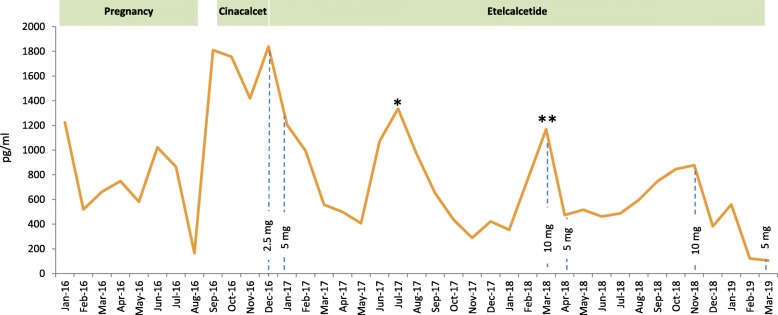
Monthly PTH levels starting with the second of pregnancy. PTH declined during pregnancy and especially with etelcalcetide. Two rebounds of PTH were caused by a *two-week and a **three-week treatment period in another unit, where etelcalcetide was not available. PTH was measured with a third generation 1–84 intact PTH assay (Elecsys PTH (1–84) assay, Roche)

At age 25 she became pregnant again. Her dialysis schedule was increased to 24 h per week (6 × 4 h), according to the current recommendations [8]. Dry weight was adjusted weekly, erythropoetin and iron supplementation were adapted as required. Oral calcitriol 0.25 μg after each dialysis session and cholecalciferol 6000 IU per week were administered and the dialysate calcium concentration was increased to 1.5 mmol/l. The patient was normophosphatemic, serum calcium was at the lower limits of normal and PTH decreased from about 1600 pg/ml to around 500 to 800 pg/ml (time-course of calcium and PTH is shown in Figs. 1 and 2). Table 1 depicts levels of serum phosphate, alkaline phosphatase and 25OH-vitamin D3 before, during and after her second pregnancy.

**Table 1:** Other relevant laboratory parameters before, during and after the second pregnancy

parameter (normal range)	3 month before second pregnancy	1. trimenon	2. trimenon	3. trimenon	3 months after pregnancy
phosphate (0.81–1.45 mmol/l)	2.33	1,08	0,94	1,24	1.71
alkaline phosphatase (35–105 U/l)	306	327	261	366	235
25OH-vitamin D3 (20–100 μg/l)	14	13	11	19	11

At 20 weeks of gestation the patient began to complain about pain in her right hip. Magnetic imaging revealed a cystic lesion in the right acetabulum, femoral neck and trochanter majus, highly suggestive of a BT.

After 36 weeks of gestation the patient gave birth to a female baby by cesarean section. Apgar score was 8/10/10, birth weight was 2755 g, and body length 47 cm. Except for a complete atrioventricular septum defect, which had already been diagnosed prenatally, the baby was healthy. The patient breastfed the neonate for five weeks.

**Fig. 3:**
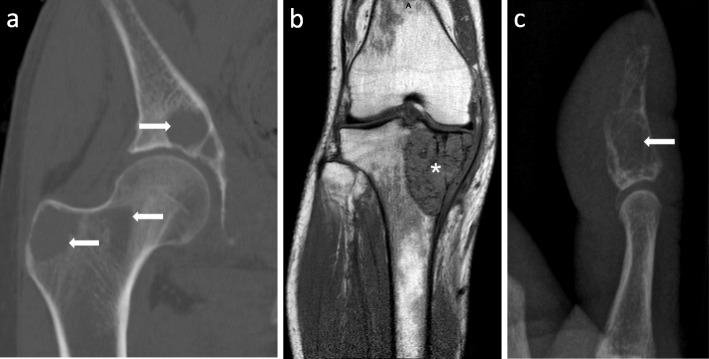
Imaging studies of selected osteolytic lesions. **a**. CT scan of the right hip. Osteolytic lesions are present in the acetabulum, femoral neck and trochanter majus (arrows). **b**. MRI scan showing a large brown tumor in the head of the right tibia (*). **c**. x-ray of the left thumb reveals osteolytic destruction of the end phalanx (arrow)

**Fig. 4:**
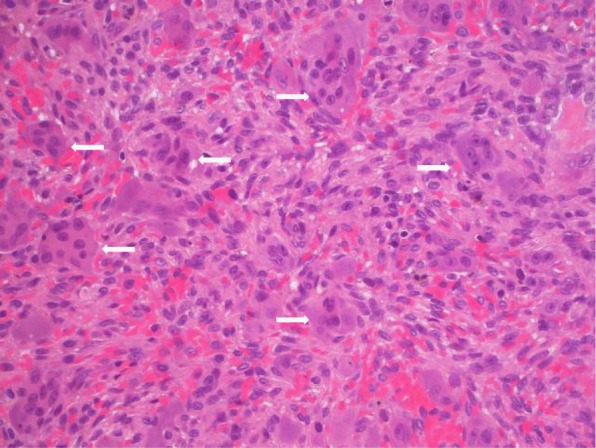
Histopathology of the brown tumor removed from the right tibia. Hematoxylin-eosin staining (400× magnification). Proliferation of mesenchymal cells with oval nuclei and eosinophilic cytoplasm. Scattered throughout the stroma are numerous osteoclast-like multinucleated giant cells containing varying numbers of vesicular nuclei (arrows)

After pregnancy, hemodialysis frequency was reduced to a conventional schedule of four hours three times weekly. PTH levels started to increase rapidly again, reaching up to 2000 pg/ml (Fig. 2). Treatment with cinacalcet with rapid dose increase from 30 to 90 mg was started, and calcitriol was continued at 0.5 μg. Both cinacalcet and calcitriol were given three times per week after the dialysis session, because the patient refused to take these medications on dialysis-free days. Further computed tomography, magnetic resonance and x-ray imaging identified multiple additional BTs of her skeleton, including the right acetabulum, the right trochanter majus, os pubis (Fig. 3a), the right tibia (Fig. 3b), several ribs, the skull and the left thumb (Fig. 3c). The patient had to be partially immobilized because of the high fracture risk. The BT in the right acetabulum and right tibia were enucleated, and the cavities were filled with autologous and homologous bone graft and an osteosynthesis of the tibia had to be performed for stabilization. Histopathology of the enucleated material confirmed the diagnosis of BTs, showing cell-rich tumor tissue with osteoclastic giant cells, multiple sideromacrophages and hemosiderin deposition (Fig. 4).

Despite treatment with cinacalcet, PTH concentration remained at around 1500 pg/ml. At that time, etelcalcetide was approved by the European Medicines Agency. Three months after delivery treatment with etelcalcetide was initiated. The initial dose of 2.5 mg after hemodialysis had to be gradually increased to 10 mg per dialysis session. Although PTH decreased to around 500 pg/ml and further on to 200 pg/ml when undergoing treatment with etelcalcetide (Fig. 2), OFC lesions did not show any sign of regression. Second enucleations of the BT in the right acetabulum and in the right tibia became necessary eight and 12 months after starting with etelcalcetide, because the bone grafts had been absorbed. In spite of sHPT being well-controlled with etelcalcetide, calcium and calcitriol supplementation, a new BT developed in the thoracic spine two years after starting etelcalcetide. A costotransversectomy on the right side of the fifth thoracic vertebral body, filling of the cavity with homologous bone graft and spondylodesis TH 4 to TH 6 was performed. Table 2 summarizes all surgical procedures performed during the course of the disease. After all these interventions the patient was fully mobile and without pain. Bone mineral density assessed by dual-energy x-ray absorptiometry in the second year after pregnancy revealed low bone mass (osteopenia) both in the lumbar spine (0.970 g/cm^2^, T-score − 1.8) and femoral necks (0.760 g/cm^2^, T-score − 2.0).

**Table 2:** Surgical procedures related to brown tumors performed during the course of the disease

Time after delivery	Procedure
3 months	enucleation of BT in the right tibia and acetabulum bone graft filling osteosynthesis
12 months	enucleation of BT right acetabulum bone graft filling
16 months	enucleation of relapsing BT right tibia and acetabulum bone graft filling osteosynthesis
19 months	enucleation of BT in the left thumb
28 months	costotransversectomy thoracic vertebra 5 osteosynthesis thoracic vertebra 4 to 6, bone graft filling

## Discussion and conclusions

This case illustrates that uncontrolled sHPT during pregnancy in a dialysis patient can have deleterious effects for the maternal skeleton.

Pregnancy, particularly multiparity, is still rare in dialysis patients. A systematic review demonstrated an increasing number of reported pregnancies from 90 cases in the period from 2000 to 2008 to 574 pregnancies in 543 women from 2000 to 2014 [9]. Increased dialysis dose goes along with higher fertility and intensified dialysis schedules, especially daily hemodialysis, quotidian long-hour dialysis or nocturnal hemodialysis, result in better maternal and fetal outcomes [10, 11].

OFC is a rare manifestation of severe pHPT and sHPT, especially in developed countries. Osteoclastic bone resorption with destruction of trabeculae is accompanied by cellular repair mechanisms that result in the accumulation of fibrous stroma and connective tissue cells along with multinucleated giant cells. The name “brown tumor” derives from the color, which is caused by hypervascularity, hemorrhage and deposits of hemosiderin. The lesions are localized in areas of intense bone resorption, preferentially in the facial skeleton but also in the clavicle, ribs and pelvic bones [4]. On imaging, they appear as lytic lesions with regular borders and thinned cortical bone, not accompanied by periosteal reaction or inflammation.

In general, BTs are three times more common in women than in men, possibly related to the large amounts of calcium and vitamin D required during pregnancy and lactation [4].

In pregnancy adaptive mechanisms are needed to cope with the calcium requirements of the developing fetus. The human fetus accretes about 30 g of calcium by term, mostly in the third trimester [12]. To meet this increased demand maternal intestinal calcium absorption more than doubles beginning in the 12th week of pregnancy, driven in part by an increase in serum calcitriol [13]. PTH is suppressed and therefore not the cause of increased calcitriol levels. Evidence from animal models suggests that prolactin or placental lactogen, and also PTH-related peptide (PTHrP) may stimulate the renal 1α-hydroxylase to produce calcitriol [12]. Although the placenta also expresses the key enzyme 1α-hydroxylase, it seems that the maternal kidneys account for most of the circulating calcitriol during pregnancy, as illustrated by an anephric woman on hemodialysis who had low calcitriol before and during her pregnancy [12].

During lactation, maternal calcium and bone metabolism must adapt to the extra demand for calcium (300–400 mg/days). The major source providing calcium during breastfeeding is bone [14]. Maternal bone mass declines during lactation by about 10% over the first six months, the losses being greatest in the trabecular skeleton [14]. The lactating breast secretes PTHrP into the systemic circulation and milk. PTHrP mobilizes skeletal calcium stores. Concomitant estrogen deficiency secondary to hypogonadotropic hypogonadism may increase bone loss [4]. Randomized clinical trials and observational studies have found that higher calcium supplementation does not reduce lactational bone density decline [15]. In the postweaning phase the skeleton is restored to its previous strength and mineral content [13].

In a pregnant dialysis patient the kidneys obviously cannot increase calcitriol synthesis. Therefore, if calcitriol and calcium are not supplemented, calcium will probably be mobilized from maternal bone. This may be aggravated by preexisting uncontrolled sHPT. In pregnancy, bone is probably particularly vulnerable to the effects of PTH, as exemplified by cases of OFC in pregnant women with pHPT. In addition, the physiologic repair mechanisms of bone in the postweaning phase may not be as effective in an ESKD patient with sHPT. Therefore, our patient may have entered the second pregnancy with an already pre-damaged and vulnerable skeleton.

What implications can be derived from normal physiology during pregnancy and from the bone and mineral derangements in CKD-MBD for the management of a pregnant dialysis patient?

First of all, pregnancy in a dialysis patient needs to be carefully planned with the medical team, not only considering blood pressure, volume or anemia management, but also avoidance or therapy of severe sHPT before entering pregnancy. On both occasions our patient did not inform the medical team about her plan to become pregnant. Pregnancy in a patient with uncontrolled sHPT should not be pursued. The first pregnancy with well controlled sHPT did not cause obvious or clinically significant damage to the maternal skeleton, whereas during the second pregnancy severe sHPT led to generalized OFC.

Second, the special fetal demands for calcium need consideration. The World Health Organization (WHO) recommends daily supplementation of 1.5 to 2 g of calcium for pregnant women after the 20th week of pregnancy, especially in those at risk for preeclampsia and in regions with low dietary calcium intake [16]. Dialysis patients are at risk for preeclampsia, and their calcium intake from milk products is usually low, because the intake of dairy products is discouraged due to their phosphate content. Therefore, oral calcium supplementation should be considered in a pregnant dialysis patient.

The WHO guidelines for antenatal care advise against routine vitamin D supplementation in pregnancy [17]. In a dialysis patient in whom renal calcitriol synthesis is absent, calcitriol supplementation - and not native vitamin D supplementation - is probably necessary to facilitate intestinal calcium absorption.

Another means of calcium supplementation in a dialysis patient is to increase the calcium dialysate concentration. Whereas a four hour bicarbonate dialysis with a dialysate calcium of 1.25 mmol /l results in a neutral calcium balance, increasing the dialysate calcium to 1.5 mmol/l provides a positive calcium balance of around 300 mg per session [18]. Whether this amount is sufficient to cover the needs of pregnancy and lactation or whether additional oral supplementation is necessary is unknown. In any case, our patient refused to take oral calcium supplements and increasing calcium in the dialysate bath seemed a plausible alternative.

All these measures, namely intensive dialysis with normalization of serum phosphate levels, calcium and calcitriol supplementation or increasing the dialysis calcium bath, will lead to a reduction in PTH levels, as observed in our patient’s second pregnancy.

Cinacalcet and etelcalcetide are calcimimetic agents, which effectively reduce PTH, calcium and phosphorous in dialysis patients [19–21]. With regard to bone turnover and histology, cinacalcet has been shown to decrease histomorphometric markers of bone turnover after six to twelve months of treatment in dialysis patients with biopsy-proven high bone turnover [3]. The package insert for cinacalcet states that cinacalcet should be used during pregnancy only if the benefits outweigh potential harms. Experimental animal studies showed cinacalcet to have no teratogenicity. Only a few case reports describe treatment with cinacalcet during pregnancy, mainly in pHPT patients to control hypercalcemia, and only for a few weeks during the third trimester [5, 22].

CKD-MBD guidelines suggest parathyroidectomy for those patients with severe hyperparathyroidism who fail to respond to pharmacological therapy [23]. Parathyroidectomy during pregnancy has to our knowledge been performed only in pHPT, but not in sHPT [24]. The current recommendation is to perform parathyroidectomy during the second trimester of pregnancy due to incomplete organogenesis in the first trimester and the risk of preterm labor in the third trimester [25]. During pregnancy PTH levels were not in the range above 800 pg/ml, where surgery is recommended and serum calcium and phosphorus were normal. For this reason, we did not consider parathyroidectomy during the second pregnancy. In the postpartal period with normalization of the dialysis schedule PTH increased rapidly despite cinacalcet (Fig. 2), and parathyroidectomy was strongly considered. We were, however, reluctant to take this step because of the fear of consequent adynamic bone disease precluding the healing of osteolytic lesions, and severe and sustaining hypocalcemia in a patient with known poor adherence in the long term.

Etelcalcetide is a novel second-generation calcimimetic given intravenously after each hemodialysis session [20]. When this new drug became available, we quickly administered it in our patient. Indeed, our patient was the first to be treated with this new second-generation calcimimetic in Europe outside clinical trials. Serum PTH levels declined with increasing doses, but rose episodically whenever the patient was treated for a short period of time in other dialysis units where etelcalcetide was not available at that time. When pharmacological treatment of sHPT to cure OFC is pursued, the precise target level of PTH, which ensures optimal bone turnover for healing, is currently unknown. During the first two years on etelcalcetide PTH levels undulated around 500 pg/ml. As shown in our patient, this level of PTH is obviously too high to allow regression of BTs. Therefore, we targeted a lower level between 100 and 200 pg/ml. Whether this PTH range allows regression of the lesions remains to be seen.

Looking at this case in retrospect, would there have been opportunities to improve patient management and outcome? Using a dialysate calcium concentration of 1.5 mmol/l in the period between the two pregnancies might have counteracted the development of severe sHPT. Secondly, parathyroidectomy before the second pregnancy would have been an option bearing in mind the possibility of postsurgical hypoparathyroidism and the development of adynamic bone disease in a young patient (potentially aggravated by her non-adherence). Thirdly, increasing the dose of calcitriol and oral calcium supplementation during pregnancy avoiding overt hypercalcemia may have been considered. Finally, a more liberal up-titration of etelcalcetide to reduce PTH accepting some degree of hypocalcemia could have been helpful.

Should breastfeeding be recommend in a dialysis patient? We are aware of only one case report on this topic [26]. Breast milk composition varies between pre- and post-dialysis samples, and post-dialysis milk is preferable for breastfeeding [26]. Under normal physiologic conditions breastfeeding puts greater stress on the skeleton than does pregnancy itself. Therefore, we suggest that in a patient with sHPT and bone disease breastfeeding should be discouraged to prevent further aggravation of bone resorption.

This case highlights the clinical problems that may arise when a dialysis patient enters pregnancy with severe sHPT. The combined effects of sHPT and pregnancy can cause OFC, requiring multiple surgical interventions. Clinical experience with usual therapeutic interventions to control PTH such as calcimimetic drugs or parathyroidectomy is practically absent. Therefore a woman on hemodialyis should be advised to get pregnant only after PTH and mineral metabolism are well controlled.

## Data Availability

Data sharing is not applicable.

## Abbreviations

BT: Brown tumor
CKD-MBD: Chronic kidney disease-associated mineral and bone disorder
ESKD: End-stage kidney disease
OFC: Osteitis fibrosa cystica
pHPT: Primary hyperparathyroidism
PTH: Parathyroid hormone
PTHrP: PTH-related peptide
sHPT: Secondary hyperparathyroidism
WHO: World Health Organization
